# The influence of degree of labelling upon cellular internalisation of antibody-cell penetrating peptide conjugates†

**DOI:** 10.1039/d2ra05274a

**Published:** 2022-09-28

**Authors:** Toni A. Pringle, Oliver Coleman, Akane Kawamura, James C. Knight

**Affiliations:** School of Natural and Environmental Sciences, Newcastle University Newcastle Upon Tyne UK james.knight2@newcastle.ac.uk; Chemistry Research Laboratory, Department of Chemistry, University of Oxford Oxford UK; Newcastle University Centre for Cancer, Newcastle University Newcastle Upon Tyne UK

## Abstract

Antibody-based agents are increasingly used as therapeutics and imaging agents, yet are generally restricted to cell surface targets due to inefficient cellular internalisation and endosomal entrapment. Enhanced cell membrane translocation of antibodies can be achieved by the covalent attachment of cell-penetrating peptides, including the HIV-1-derived transactivator of transcription (TAT) peptide. This study evaluated the cellular internalisation properties of five anti-HER2 Herceptin–TAT conjugates with degrees of TAT labelling (DOL_TAT_) ranging from one to five. Herceptin–TAT conjugates were synthesised *via* a strain-promoted alkyne–azide cycloaddition reaction, characterised by UV-vis spectroscopy, MALDI-TOF, and gel electrophoresis, then radiolabelled with zirconium-89 to permit measurement of cellular internalisation by gamma counting. [^89^Zr]Zr–DFO–Her–TAT_(0–5)_ conjugates were isolated in high radiochemical purity (>99%) and exhibited high stability in murine and human serum over 7 days at 37 °C. Significant increases in cellular internalisation were observed for [^89^Zr]Zr–DFO–Her–TAT conjugates with DOL_TAT_ values of 2 and above in SKBR3 (high HER2) cells over 48 h, in contrast to low-level non-specific uptake in MDA-MB-468 (low HER2) cells that did not increase over time. Notably, [^89^Zr]Zr–DFO–Her–TAT conjugates with DOL_TAT_ of 3, 4, and 5 reached uptake values in SKBR3 cells of 5, 6, and 8% of the applied dose at 48 h respectively, representing 9, 10, 14-fold increases relative to the TAT-free control conjugate, [^89^Zr]Zr–DFO–Her–TAT_(0)_.

## Abstract 2

A systematic investigation into the influence of degree of labelling of antibody-cell penetrating peptide conjugates upon cellular internalisation.

## Graphical abstract

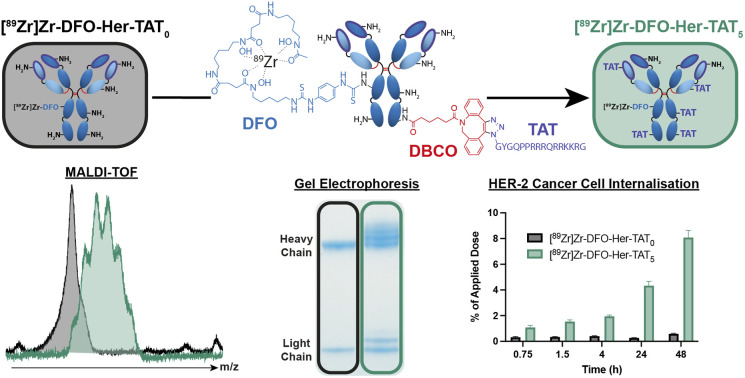

## 1. Introduction

Monoclonal antibodies (mAbs) are valuable tools in the era of precision medicine due to their ability to bind an array of clinically relevant cell surface proteins with high binding specificity, selectivity, and affinity.^1^ Most mAb therapies are based on direct disruption of cancer cell activity (*e.g.* inhibition of growth factor receptor signalling) or immune activation (*e.g.* antibody-dependent cell-mediated cytotoxicity, ADCC), however they are also commonly modified with cytotoxic cargoes to yield antibody–drug conjugates (ADCs) with high therapeutic indices. A critical determinant of ADC efficacy is sufficient internalisation in target cell populations to ensure rapid intracellular delivery of the cytotoxin and minimise toxicity arising from extracellular delivery. It is also worth noting the promising utility of mAb-based companion diagnostics in positron emission tomography (immunoPET) investigations for aiding patient selection and enabling rapid assessment of treatment response. However, this otherwise attractive imaging modality is similarly limited by inefficient internalisation and endosomal entrapment of mAb-derived probes which restricts the scope of this application to a small subset of clinically relevant cell surface biomarkers, in contrast to the rich pool of biomarkers located inside cells.^2–4^

Cell-penetrating peptides (CPPs, also known as protein transduction domains) are short peptides (typically 10 to 30 amino acids) capable of enhancing the cellular internalisation of either covalently conjugated or co-delivered cargos, including mAb-based imaging and therapeutic agents,^5–7^ mostly by energy-dependant endocytosis mechanisms.^8,9^ Notably, the TAT peptide (GRKKRRQRRRPPQGYG) derived from the transactivator of transcription protein of the HIV-1 virus has been widely applied as a CPP, including in the development of antibody–CPP conjugates which have emerged in recent years.^3^ For example, an immunoPET agent based on a TAT-functionalised anti-γH2AX antibody has been developed to monitor DNA damage response signalling during tumorigenesis and cancer therapy.^10,11^ Furthermore, two recent independent studies by Tietz *et al.* and Sauter *et al.* reported significantly enhanced cytosolic delivery of mAb-derived agents *via* the integration of multimeric CPPs (trimeric and tetrameric, respectively), highlighting the importance of several CPP-related parameters, including degree of labelling, concentration, spatial proximity, and local charge density, upon cellular internalisation.^12,13^ Each study also reported that non-specific cell uptake increased at higher CPP : mAb ratios which indicates the need to carefully optimise this key parameter to maximise specific uptake in target cell populations.^14^

Here, we describe the synthesis, characterisation, and *in vitro* cellular internalisation properties of five anti-HER2 Herceptin–TAT conjugates with degrees of TAT labelling (DOL_TAT_) ranging from 1–5 with the aim to facilitate the future optimisation of CPP-modified antibody-based pharmaceutics. To this end, a bioconjugation strategy based on strain-promoted alkyne–azide cycloaddition (SPAAC)^2^ that enables UV/vis-based DOL_TAT_ determination was utilised that distinguishes the Herceptin–TAT conjugates from those previously reported and synthesised using an amine-to-sulfhydryl (sulfo-SMCC) crosslinker.^15^

## 2. Experimental procedures

### 2.1. Materials and methods

All reagents were purchased from Fisher Scientific unless otherwise stated and used without further purification. Water was deionized using a Select Fusion ultrapure water deionisation unit (Suez) and had a resistance of >18.2 MΩ cm^−1^ at 25 °C. Protein concentration measurements were obtained using a NanoDrop One^C^ Microvolume UV-vis Spectrophotometer (NanoDrop Technologies, Inc.). MALDI-TOF mass spectrometry measurements were taken on a Bruker Microflex LRF. Radioactivity measurements were obtained using a CRC-25 Dose Calibrator (Capintec, Inc.) or a Wizard 2480 Gamma Counter (PerkinElmer). Radioimmunoconjugate synthesis and serum stability studies were monitored by instant thin-layer chromatography using glass microfiber chromatography paper (iTLC-SA, Agilent). Radio-iTLC strips were measured by autoradiography (Amersham Typhoon Bioimager, GE) and analysed using ImageQuant software (GE Healthcare). pH measurements were determined using pH indicator paper (Merck Millipore) or a pH Spear electrode (Eutech Instruments).

### 2.2. Conjugation of DBCO–STP ester to mAb

Between 2 and 3 mg of trastuzumab (Herceptin®, Her) was dissolved in 0.1 M NaHCO_3_ (pH 9, 500 μL) then passed through a pre-rinsed 30 kDa molecular weight cut-off 0.5 mL centrifugal filter (Amicon) at 12 000 × *g* for 10 min and washed three times (3 × 500 μL) to remove excipients. The antibody solution was adjusted to 5 mg mL^−1^ with 0.1 M NaHCO_3_ (pH 9) based on UV-vis absorbance measurements. A 1, 2, 3, 4, 5, 6 or 7-fold molar excess of DBCO–STP ester (Click Chemistry Tools) was added from a 5 mM stock solution in DMSO to the antibody solution and the reaction mixture was incubated at 25 °C for 2 h with gentle shaking (450 rpm). Centrifugal filtration using the previously described method enabled isolation of purified Her–DBCO_(1–7)_ conjugates in PBS (450 μL, pH 7.2). Absorbances of Her–DBCO_(1–7)_ conjugates at 280 and 309 nm were measured by UV-vis spectroscopy to determine the degree of DBCO labelling and antibody concentration (see Section 2.4).

### 2.3. SPAAC conjugation of TAT–N_3_ to mAb–DBCO

Azide-modified TAT (1 mM in PBS, TAT–N_3_ = GRKKRRQRRRPPQGYG–hA(N_3_), Cambridge Peptides) was added in 10-fold molar excess to a 5 mg mL^−1^ solution of Her–DBCO in PBS (pH 7.2) then incubated at 25 °C for 2 h with gentle shaking (450 rpm). The product, Her–TAT, was purified by passing the reaction mixture through a pre-rinsed 30 kDa molecular weight cut-off 0.5 mL centrifugal filter at 12 000 × *g* for 10 minutes and washing three times (3 × 500 μL) with PBS (pH 7.2). The absorbances of Her–TAT_(1–5)_ conjugates at 280 and 309 nm were measured by UV-vis spectroscopy to determine the degree of TAT labelling and antibody concentration (see Section 2.4).

### 2.4. Degree of labelling determination

The average number of DBCO moieties attached to each antibody (DOL_DBCO_) was calculated using the following equation:
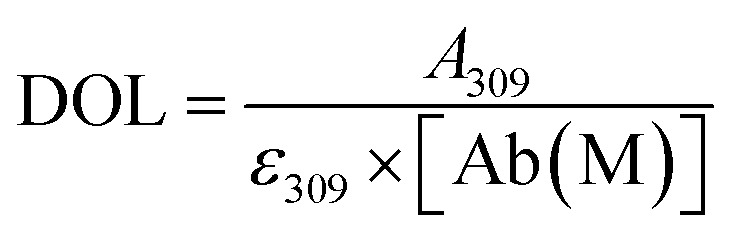
where
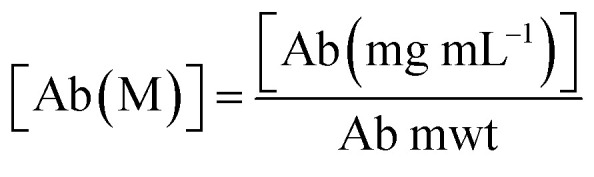
and
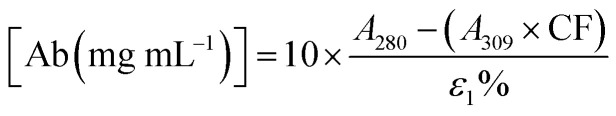
CF = correction factor (*A*_280_/*A*_309_), *ε*_309_ = molar attenuation coefficient at 309 nm, *ε*_1_% = percent molar attenuation coefficient for a 10 mg mL^−1^ IgG solution.

Following the SPAAC reaction between TAT–N_3_ and Her–DBCO and purification by size exclusion chromatography (Section 2.5), the average number of residual unreacted DBCO moieties attached to each antibody (DOL_DBCO*_) was determined by the same approach. The average number of TAT peptides attached to each DBCO-modified antibody (DOL_TAT_) was determined by subtraction of DOL_DBCO*_ from the initial DOL_DBCO_ (DOL_TAT_ = DOL_DBCO_ − DOL_DBCO*_).

### 2.5. Size exclusion chromatography

Crude Her–TAT_(1–5)_ conjugates were purified by Sephadex-G50 (Sigma-Aldrich) size exclusion chromatography in a 1 mL column, eluting with 100 μL fractions of PBS (pH 7.2). Her–TAT_(1–5)_ conjugates were eluted between fractions 8–12. Fractions were combined to achieve the desired DOL_TAT_ value of 0–5.

### 2.6. DFO attachment

A 10-fold molar excess of *p*-SCN–Bn–deferoxamine (*p*-SCN–Bn–DFO, Macrocyclics) from a 2 mg mL^−1^ stock solution in DMSO was added to Her–TAT_(0–5)_ (500 μg) in 0.1 M NaHCO_3_ (pH 8.5, 100 μL). The reaction mixture was incubated at 37 °C for 1 h with gentle shaking (450 rpm). The DFO-modified antibody conjugates were then purified by centrifugal filtration as previously described and adjusted to 2 mg mL^−1^ with PBS (pH 7.2) in preparation for radiolabelling with zirconium-89 (^89^Zr).

### 2.7. SDS-PAGE

Her–TAT_(0–5)_ conjugates were analysed by sodium dodecyl sulfate-polyacrylamide gel electrophoresis (SDS-PAGE) under reducing conditions. Antibody samples (1–2 μL, 1 mg mL^−1^) were prepared by the addition of NuPAGE 4× LDS sample buffer (2.5 μL), NuPAGE 10× Sample Reducing Agent (1 μL), and deionised water (4.5–5.5 μL) to a total volume of 10 μL. The resulting solutions were incubated at 70 °C for 10 min at 450 rpm. Protein samples and molecular weight standards (Thermo Scientific PageRuler Unstained Broad Range Protein Ladder, 5 μL) were loaded on a 10-well protein gel (4–12% Bis–Tris) and run for 45 minutes at 200 V in NuPAGE MOPS SDS running buffer. The gel was subsequently washed three times in deionised water (200 mL, 5 min) then stained with SimplyBlue SafeStain (50 mL, Invitrogen) for 1 h. Destaining was performed by washing three times in deionised water (200 mL, 1 h).

### 2.8. MALDI-TOF

A saturated solution of α-cyano-4-hydroxycinnamic acid (CHCA, 20 mg) in acetone (500 μL) was prepared as Mix 1. A precursor saturated solution of CHCA (20 mg) in 70% acetonitrile with 5% formic acid (500 μL) was prepared alongside a saturated solution of 2,5-dihydroxybenzoic acid (DHB, 20 mg, Sigma-Aldrich) in 70% acetonitrile with 0.1% trifluoroacetic acid (500 μL). Solutions were prepared at room temperature and vortexed thoroughly for 60 seconds before use. DHB (100 μL) and CHCA (100 μL) solutions were then combined to prepare Mix 2. Mix 1 (0.5 μL) was spotted onto a polished steel target plate (Bruker) and evaporated quickly to leave a thin layer of CHCA. A 0.5 μL aliquot of protein sample (typically 10 μM in PBS) was spotted directly onto the layer. Then, 0.5 μL of Mix 2 was added to the liquid droplet and allowed to dry. Where required, antibody samples were reduced by incubation with dithiothreitol (DTT, 10 mM) at 60 °C for 30 min.

A Bruker Microflex LRF was used to acquire the MALDI-TOF-MS data, in linear positive mode (laser 60 Hz, ion source 1: 19.5 kV, ion source 2: 18.15 kV, lens: 7.00 kV, pulsed ion extraction 240 ns, detector gain 2850 V). Data were processed using Bruker flexAnalysis v3.4.

### 2.9. Radiolabelling

Zirconium-89 in 1 M oxalic acid (∼5–10 MBq, PerkinElmer) was adjusted to pH 7 by the addition of 1 M Na_2_CO_3_. The solution was briefly shaken and left at room temperature for 2–3 min until the evolution of CO_2(g)_ had stopped. The neutralised ^89^Zr solution was added to DFO–Her–TAT_(0–5)_ (∼100 μg, 2 mg mL^−1^) and then incubated at 25 °C for 1 h with gentle shaking (450 rpm). The radiolabelling efficiency and radiochemical purity was assessed by radio-instant thin layer chromatography (radio-iTLC) using 50 mM EDTA (pH 5.5) as the mobile phase, where ^89^Zr-labelled antibody conjugates remained at the origin (*R*_f_ = 0) and free ^89^Zr migrated to the top of the iTLC strip (*R*_f_ = 0.8–1).^16^ Radioimmunoconjugates were used in *in vitro* assays when RCP was >99%.

### 2.10. Serum stability

[^89^Zr]Zr–DFO–Her–TAT conjugates (1.4 MBq, 12.2 μg, 7 μL) were added to 500 μL of human serum (Sigma-Aldrich, H4522), mouse serum (Sigma-Aldrich, M5905), or PBS (pH 7.2) in triplicate. The resulting mixtures were incubated at 37 °C for 7 days with gentle shaking (450 rpm) and radioimmunoconjugate stability was assessed at 24 h intervals by radio-iTLC as previously described.

### 2.11. Cell culture

The human breast cancer cell lines SKBR3 (high HER2 expression) and MDA-MB-468 (low HER2 expression) were obtained from American Type Culture Collection (ATCC). Cell lines were maintained in RPMI (Sigma-Aldrich, R8758) and DMEM (Gibco, 41965039) medium, respectively. Media were supplemented with 10% foetal bovine serum (FBS, Sigma-Aldrich, F9665), 2 mM l-glutamine, penicillin (100 units per mL), and streptomycin (0.1 mg mL^−1^, Sigma-Aldrich, G6784), and maintained in a 5% CO_2(g)_ humidified atmosphere at 37 °C. Cells were harvested and passaged when they reached 85–95% confluency using trypsin–EDTA solution (Sigma-Aldrich, T4049). The cumulative length of culture was less than 6 months following retrieval from liquid nitrogen storage. Cells were tested for the absence of mycoplasma at regular intervals.

### 2.12. Cell internalisation assay

SKBR3 and MDA-MB-468 cells were seeded in 0.5 mL media in 24-well plates at the necessary density to achieve 50 000 cells per well at each time point of the experiment. To each well, 1.47 μL was added from respective [^89^Zr]Zr–DFO–Her–TAT_(0–5)_ stock solutions (0.21 ± 0.02 MBq, 2.42 μg) diluted in 0.5 mL of media. At 0.75, 1.5, 4, 24 and 48 h incubation, the supernatant media (free fraction) was transferred to allocated counting tubes and combined with 0.5 mL of PBS (pH 7.2) used to wash the cells. Cells were then incubated in cold 0.1 M glycine HCl (0.5 mL, pH 2.5, Sigma-Aldrich) for 10 min and this solution was then transferred to a separate series of counting tubes (membrane-bound fraction) followed by the addition of a 0.5 mL PBS (pH 7.2) cell wash. Lastly, RIPA lysis and extraction buffer (0.1 mL, Thermo Scientific) was added to each well and plates were placed on ice for 5 min before transferring the lysed cell solutions (internalised fraction) to a third series of counting tubes, followed by the addition of a 0.5 mL PBS (pH 7.2) cell wash. The activity in each counting tube was measured as counts per minute (cpm) on a gamma counter, converted to activity units (MBq) using recent calibration data, and decay corrected to the start of the experiment.

### 2.13. Statistical analysis

All statistical and regression analyses were performed using GraphPad Prism v9 (GraphPad Software, San Diego, CA, USA). A confidence interval of 95% (*P* < 0.05) was considered statistically significant. Unpaired *t*-tests or two-way ANOVA followed by Šídák's multiple comparisons test was used to compare multiple groups. All data was obtained in at least triplicate, and results were reported and graphed as mean ± standard deviation, unless stated otherwise. Statistical significance is shown by asterisks where ns = not significant, * = *p* < 0.05, ** = *p* < 0.01, *** = *p* < 0.001, **** = *p* < 0.0001.

## 3. Results and discussion

### 3.1. Synthesis and characterisation

**Fig. 1:**
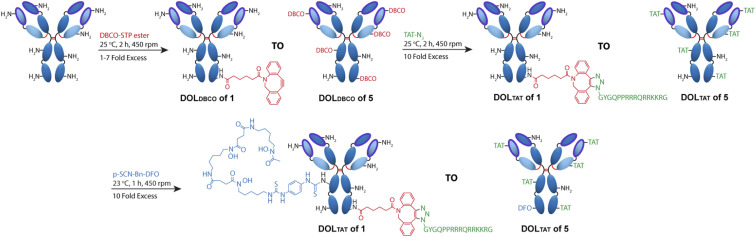
Scheme depicting the 3-step synthesis of DFO–Her–TAT_(0–5)_. Lysine residues distributed throughout the antibody (depicted as amine groups) were conjugated with dibenzocyclooctyne (DBCO) *via* a sulfo-tetrafluorophenol (STP) ester. Degrees of labelling of DBCO (DOL_DBCO_) between 1 and 7 were achieved using increasing molar excesses of DBCO–STP ester. Her–DBCO_(1–7)_ then underwent a strain promoted [3 + 2] alkyne–azide coupling (SPAAC) reaction with an excess of azide modified TAT (TAT–N_3_) to produce conjugates with DOL_TAT_ between 1 and 5 (Her–TAT_(1–5)_). Lysine residues within the antibody were further modified with desferroxamine (DFO) *via* an isothiocyanate to produce DFO–Her–TAT_(1–5)_.

Antibody–CPP conjugates (DFO–Her–TAT_(0–5)_) were prepared by the three-step synthetic pathway outlined in Fig. 1. First, dibenzocyclooctyne-4-sulfo-2,3,5,6-tetrafluorophenol (DBCO–STP) ester was added in 0, 1, 2, 3, 4, 5, 6, or 7-fold molar excess to Herceptin to facilitate acylation-based conjugation of DBCO to the ε-amino groups of lysine residues in yields typically between 60–90% after purification (see Table S1† for individual reaction yields). Her–DBCO_(0–7)_ conjugates were isolated with increasing degrees of DBCO labelling (DOL_DBCO_) consistent with the reaction stoichiometry (Table 1). Her–DBCO_(1–5)_ conjugates then underwent strain-promoted [3 + 2] alkyne–azide cycloaddition (SPAAC) reactions with a 10-fold molar excess of TAT–N_3_, yielding Her–TAT conjugates with DOL_TAT_ in close alignment with the DOL_DBCO_ of the Her–DBCO conjugate used as a precursor in each case (Table 1, Fig. S1 and S2†). Purified Her–TAT conjugates were isolated in yields typically between 80 and 90% (Table S1†).

**Table 1:** DOL_DBCO_ values following reaction of Herceptin and DBCO–STP ester (1–7-fold molar excess), and DOL_TAT_ values following reaction of Her–DBCO_(1–7)_ and TAT–N_3_ (10-fold molar excess)

Molar excess of DBCO–STP ester	DOL_DBCO_	DOL_TAT_
1	1.24 ± 0.46 (*n* = 4)	0.75 ± 0.55 (*n* = 4)
2	2.04 ± 0.26 (*n* = 4)	1.60 ± 0.25 (*n* = 4)
3	2.63 ± 0.13 (*n* = 3)	2.13 ± 0.09 (*n* = 3)
4	3.60 ± 0.43 (*n* = 5)	2.88 ± 0.20 (*n* = 4)
5	4.27 ± 0.38 (*n* = 6)	3.33 ± 0.36 (*n* = 4)
6	4.89 ± 0.38 (*n* = 3)	3.72 ± 0.24 (*n* = 3)
7	5.53 ± 0.64 (*n* = 4)	4.07 ± 0.51 (*n* = 4)

**Fig. 2:**
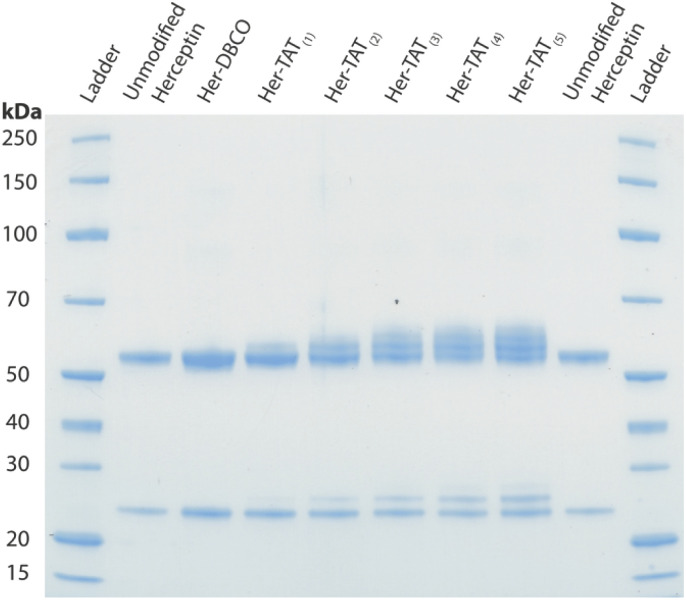
SDS-PAGE of unmodified Herceptin, Her–DBCO, and Her–TAT_(1–5)_ conjugates acquired under reducing conditions.

**Fig. 3:**
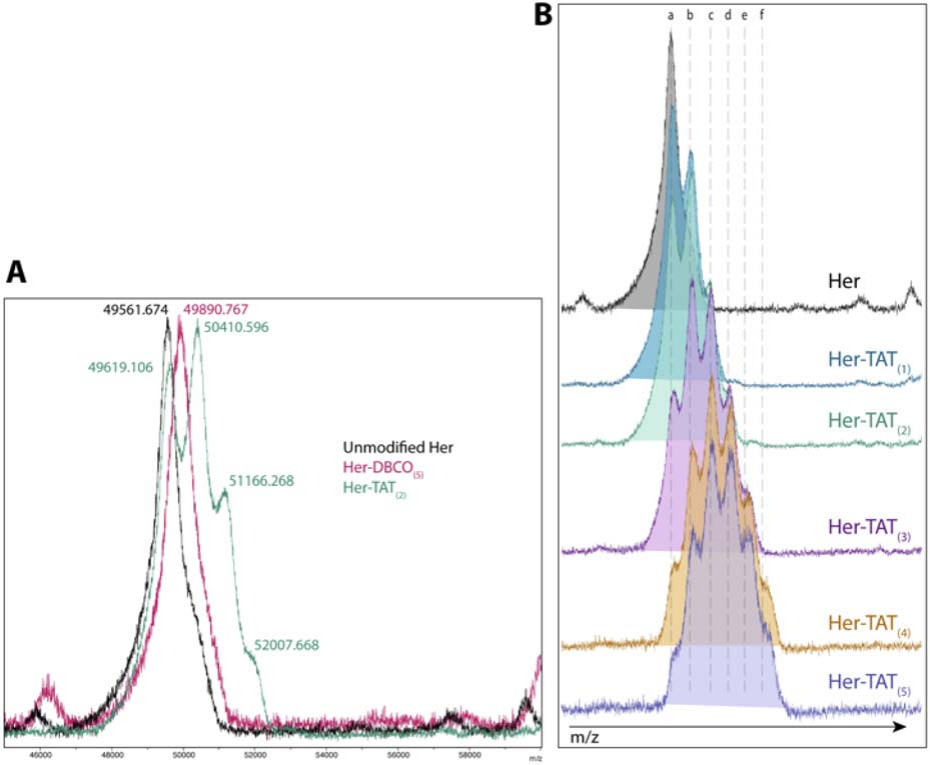
MALDI-TOF spectra of Her–TAT conjugates. (A) Unmodified Herceptin (black), Her–DBCO_(5)_ (pink) and Her–TAT_(2)_ (green). The *m*/*z* for each peak is labelled and corresponds to [M + 3]. (B) Unmodified Herceptin (black) and Her–TAT conjugates with DOL_TAT_ of 1–5. Key: *a* = 49 672.7 ± 65.6, *b* = 50 437 ± 29.2, *c* = 51 224.3 ± 66.6, *d* = 52 032.8 ± 28.4, *e* = 52 801.7 ± 11.8, *f* = 53 613.4 ± 91.6 *m*/*z* [M + 3].

SDS-PAGE analysis of Her–TAT_(1–5)_ conjugates in reducing conditions revealed additional bands above the light and heavy chains of the mAb conjugates compared to unmodified Herceptin (Fig. 2 and S3†) with spacing and multiplicity consistent with the mass of the TAT peptide and DOL_TAT_ of each sample. Similarly, MALDI-TOF analysis of Her–TAT_(1–5)_ conjugates showed multiple peaks with a spacing of 2342.44 ± 121.15 Da (*n* = 17) corresponding to the attachment of both DBCO moieties and TAT peptides (combined molecular weight: 2439 Da; Fig. 3 and Table S2†).

MALDI-TOF offered an independent method to cross-validate DOL_TAT_ values determined by UV-vis spectroscopy based on measurement of the relative peak intensity in each sample. DOL_TAT_ values ascertained by these two different methods were generally in close agreement (Table S5†). Lastly, the conjugation of ^89^Zr chelator, DFO, was achieved using an isothiocyanate-modified analogue, *p*-SCN–Bn–DFO, resulting in lysine-directed thiourea formation and generation of DFO–Her–TAT_(0–5)_ conjugates in 82.58 ± 10.99% (*n* = 9) yield.

### 3.2. Radiolabelling and serum stability

**Fig. 4:**
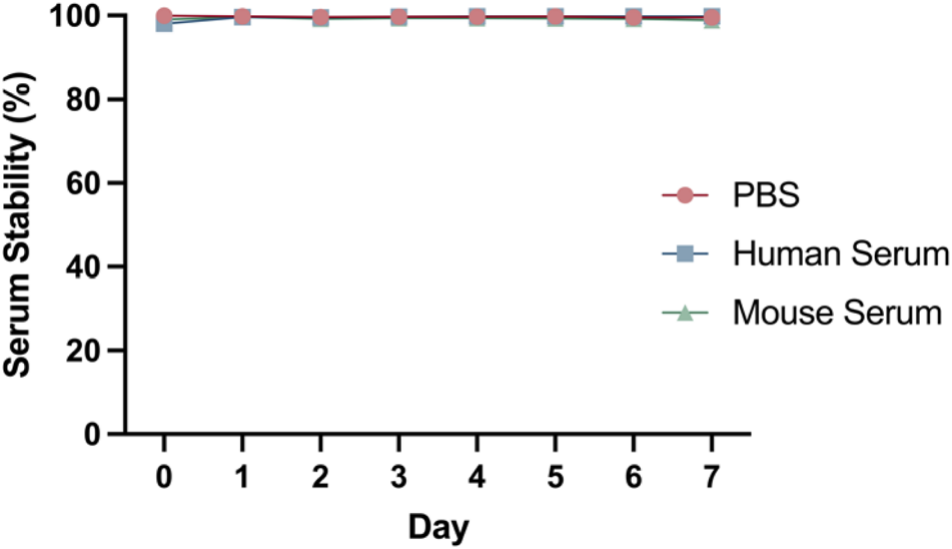
Representative serum stability graph showing the percentage of intact [^89^Zr]Zr–DFO–Her–TAT_(2)_ conjugate in PBS (red), human serum (blue) and mouse serum (green) over 7 days at 37 °C, determined by radio-iTLC.

DFO–Her–TAT_(0–5)_ conjugates were ^89^Zr radiolabelled with radiolabelling efficiencies of 99.9 ± 0.1% (*n* = 7) as determined by radio-iTLC (Fig. S4†). The DOL_TAT_ was found to have no effect upon radiolabelling efficiency. The stability of [^89^Zr]Zr–DFO–Her–TAT conjugates remained very high (>98%) in PBS, mouse serum, and human serum over 7 days at 37 °C with no evidence of radiometal dissociation (Fig. 4).

### 3.3. Cellular internalisation

**Fig. 5:**
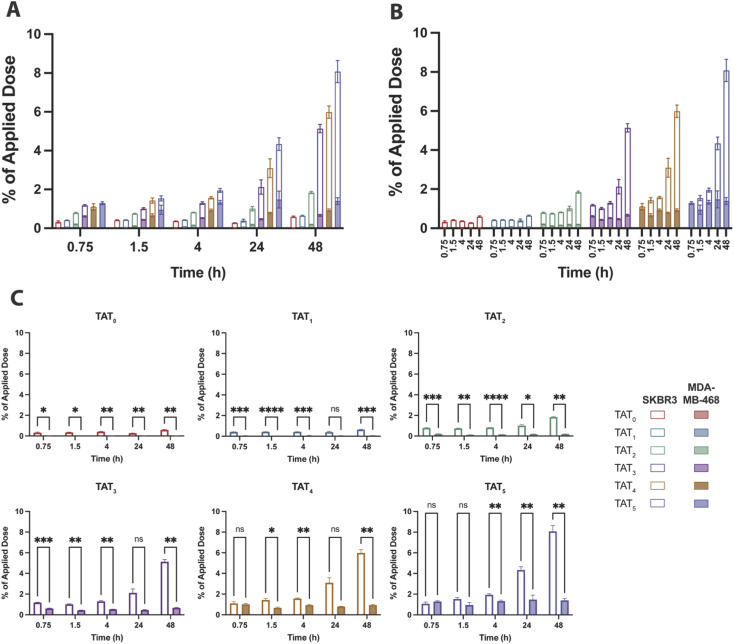
Percentage of the applied dose that internalised in SKBR3 (hollow bars) and MDA-MB-468 (filled bars) cells for each [^89^Zr]Zr–DFO–Her–TAT conjugate. (A) Overlaid internalised dose of each conjugate in SKBR3 and MDA-MB-468 cells at each time point grouped by time point. (B) Overlaid internalised dose of each conjugate in SKBR3 and MDA-MB-468 cells at each time point grouped by DOL_TAT_. (C) Individual graphs for the percentage of applied dose internalised for each [^89^Zr]Zr–DFO–Her–TAT conjugate in SKBR3 and MDA-MB-468 cells. Error bars represent standard deviation.

The baseline control conjugate without TAT, [^89^Zr]Zr–DFO–Her_(0)_, had low uptake in SKBR3 (high HER2) cells that marginally increased over time to 0.58 ± 0.04% of the applied dose at 48 h and remained consistently higher than in MDA-MD-468 (low HER2) cells which showed no significant uptake (Fig. 5). A similar uptake profile was observed for the radioimmunoconjugate modified with a single TAT, [^89^Zr]Zr–DFO–Her–TAT_(1)_, which revealed no enhancement of internalization in either cell line relative to the baseline conjugate, indicating that a DOL_TAT_ > 1 is needed to increase uptake (Fig. S5†).

In contrast, [^89^Zr]Zr–DFO–Her–TAT_(2)_ was internalized in SKBR3 cells to a greater extent than the TAT-free control at each time interval and achieved an uptake value of 0.78 ± 0.03% at 0.75 h that steadily increased to 1.01 ± 0.11 and 1.84 ± 0.05% at 24 and 48 h, respectively. Greater increases in internalization were observed for [^89^Zr]Zr–DFO–Her–TAT conjugates with DOL_TAT_ of 3, 4, and 5 which reached uptake values of 2.12 ± 0.40, 3.10 ± 0.48 and 4.34 ± 0.32% at 24 h, and later increased to 5.14 ± 0.22, 5.99 ± 0.32 and 8.08 ± 0.57% respectively at 48 h. Relative to the TAT-free control at 48 h, 1.1, 3.1, 8.7, 10.1 and 13.7-fold increases were obtained for conjugates with DOL_TAT_ values of 1, 2, 3, 4, and 5, respectively.

Notably, Sauter *et al.* measured internalisation of a Herceptin derivative modified with a tetrameric analogue of TAT (tTAT) in SKBR3 cells over 4 h and reported a ∼2.4-fold increase, in contrast to a ∼4.5-fold increase in this study for [^89^Zr]Zr–DFO–Her–TAT_(4)_ at 4 h. Non-specific TAT-mediated uptake was evident in MDA-MB-468 cells for [^89^Zr]Zr–DFO–Her–TAT conjugates with DOL_TAT_ ≥ 3 that did not increase over time but increased significantly towards higher DOL_TAT_ values (Fig. S6†). The average non-specific contribution was 0.67 ± 0.05, 0.93 ± 0.07 and 1.40 ± 0.17% of applied dose at 48 h for conjugates with DOL_TAT_ values of 3, 4, and 5, respectively.

The steady time profile of internalization in this low HER2 cell line suggests that this uptake is being offset by externalization which may occur *via* a mechanism proposed by Rayne *et al.* that describes externalization of TAT by phosphatidylinositol-(4,5)-bisphosphate on the inner leaflet of the cell membrane.^17^

## 4. Conclusions

This study has examined the relationship between DOL_TAT_ and cellular internalisation over time using a clinically relevant model based on Herceptin. The isolation of Herceptin–TAT conjugates with well-defined DOL_TAT_ values between 1 and 5 was readily achieved in high yields using a synthetic route based on strain-promoted alkyne–azide cycloaddition. For Herceptin conjugates with DOL_TAT_ values ≥ 2, significant enhancements in internalisation relative to a TAT-free Herceptin control were observed in HER2 positive SKBR3 cells but not in HER2 negative MDA-MB-468 cells, indicating HER2 specific uptake in cells. The extent of this enhancement was most prominent after 48 h incubation and increased towards the highest DOL_TAT_, although conjugates with DOL_TAT_ values of 2 and 3 showed a lower proportion of uptake in HER2 negative cells which suggests that TAT modification in this range may represent an optimal balance between total uptake and specificity. The significant increases in cellular internalisation observed in this study will be of interest to the drug development and molecular imaging communities as it may serve to increase the therapeutic indices of ADCs and expand the scope of available imaging biomarkers for immunoPET.

The systematic nature of this investigation has yielded data that contributes to our understanding of the influence of CPPs upon the cellular internalisation characteristics of antibodies. A limitation of this study, however, is its examination of just one of a great multitude of CPP candidates, and while TAT was selected on the basis of its prominent application in biomedical research over recent decades it is likely that other CPPs (*e.g.* cyclic- and multimeric-TAT,^12,13,18^ r9,^19^ CPP12 (ref. 20)) may yield even greater enhancements in internalisation and pharmacological properties,^21^ and the optimisation of this next-generation of antibody–CPP conjugates would benefit from a similar investigation to that described herein. With the principal aim of developing a facile, modular strategy capable of enhancing the therapeutic index of antibody-based drugs, future work will examine the applicability of these findings to other clinically relevant antibodies and ADCs, and systematically assess the impact of DOL_TAT_ upon key factors, including cellular internalisation mechanisms, immunoreactivity, pharmacokinetics, and biodistribution in preclinical models.

## Conflicts of interest

There are no conflicts to declare.

## Supplementary Material

RA-012-D2RA05274A-s001
