# Supplementary material for: The influence of degree of labelling upon cellular internalisation of antibody-cell penetrating peptide conjugates
Source: RSC Adv. 2022 Sep 28;12(43):27716–22. doi: 10.1039/d2ra05274a (PMC9517169; doi:10.1039/d2ra05274a)
Supplement: RA-012-D2RA05274A-s001 [file RA-012-D2RA05274A-s001.pdf]

Electronic Supplementary Material (ESI) for RSC Advances.
This journal is © The Royal Society of Chemistry 2022

Supporting Information

The Influence of Degree of Labelling upon Cellular Internalisation of Antibody-Cell Penetrating Peptide Conjugates

Toni A. Pringle[a], Oliver Coleman[a], Akane Kawamura[a,b], James C. Knight[a,c,*]

[a.] *School of Natural and Environmental Sciences, Newcastle University, Newcastle Upon Tyne, United Kingdom.*
[b.] *Chemistry Research Laboratory, Department of Chemistry, University of Oxford, Oxford, United Kingdom*
[c.] *Newcastle Centre for Cancer, Newcastle University, Newcastle upon Tyne, United Kingdom*

SUPPLEMENTARY FIGURES

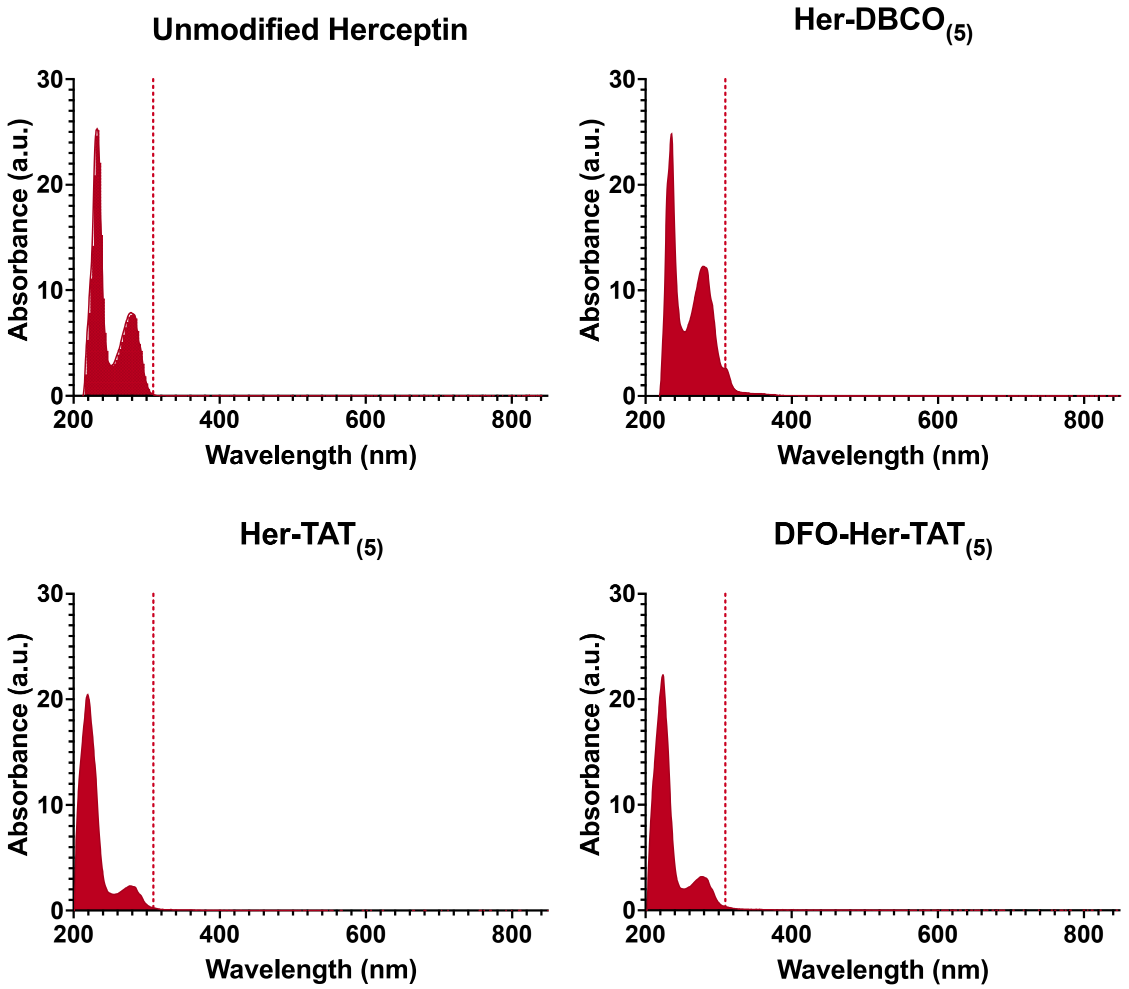

Figure S1: *UV-Vis spectra of unmodified Herceptin, Her-DBCO$_{(5)}$, Her-TAT$_{(5)}$ and DFO-Her-TAT$_{(5)}$ conjugates. Red dotted line shows 309 nm.*

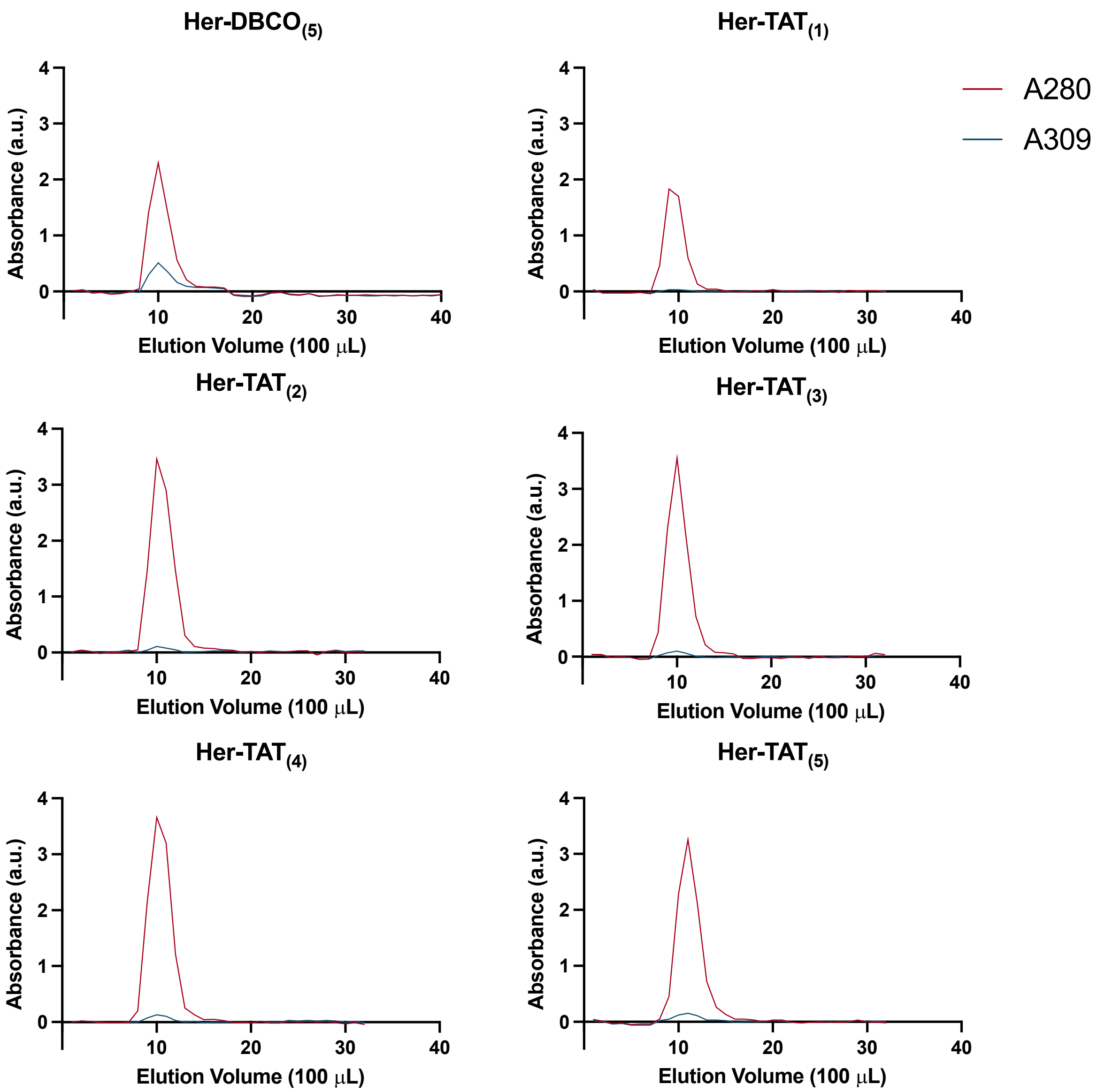

Figure S2: *UV-Vis analysis (measuring A_{280} and A_{309}) of size exclusion chromatograph fractions of Her-DBCO and Her-TAT$_{(1\text{-}5)}$ conjugates.*

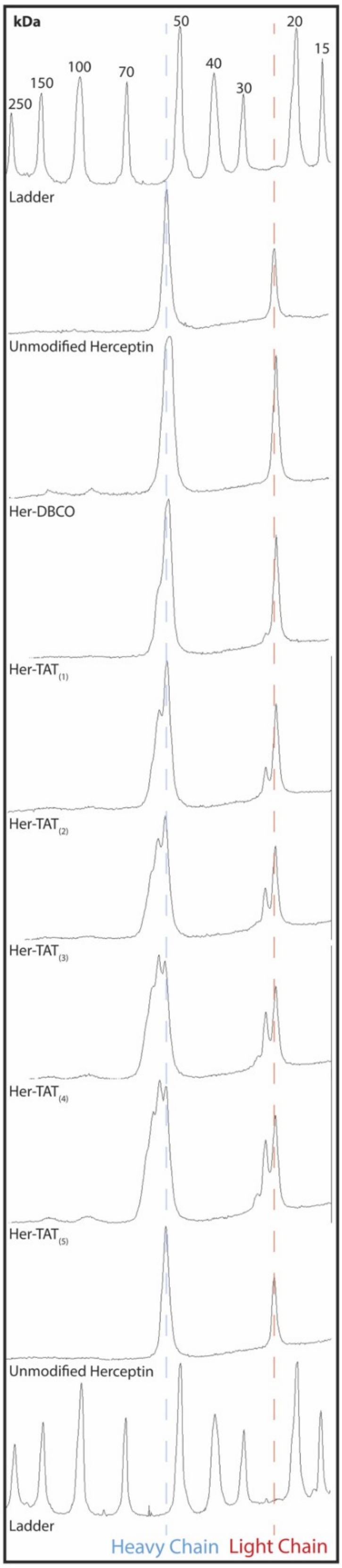

Figure S3: *Reduced SDS-PAGE densitometry analysis of unmodified Herceptin, Her-DBCO and Her-TAT$_{(1\text{-}5)}$ conjugates using ImageJ software. Key at the top depicts masses of peaks in the protein ladder. Unmodified heavy chain (blue) and light chain (red) are highlighted by dashed lines.*

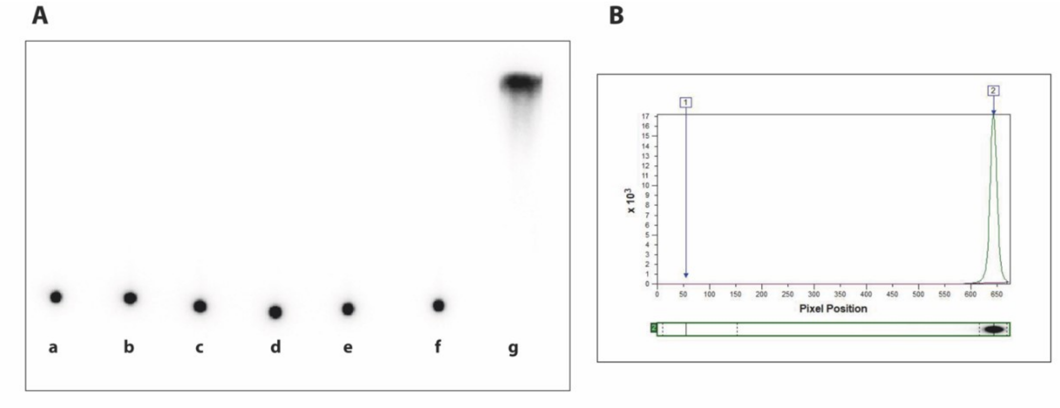

Figure S4: *Radiolabelling efficiency determination of [^{89}Zr]Zr-DFO-Her-TAT$_{(0\text{-}5)}$ conjugates. (A) Radio-iTLC of [^{89}Zr]Zr-DFO-Her (a), [^{89}Zr]Zr-DFO-Her-TAT$_{(1)}$ (b), [^{89}Zr]Zr-DFO-Her-TAT$_{(2)}$ (c), [^{89}Zr]Zr-DFO-Her-TAT$_{(3)}$ (d), [^{89}Zr]Zr-DFO-Her-TAT$_{(4)}$ (e), [^{89}Zr]Zr-DFO-Her-TAT$_{(5)}$ (f), "free" ^{89}Zr control (g). (B) Representative analysis of radio-iTLC for [^{89}Zr]Zr-DFO-Her-TAT$_{(0\text{-}5)}$ conjugates.*

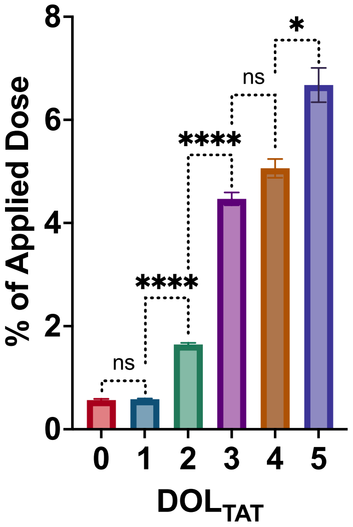

Figure S5: *Percentage of applied dose of [^{89}Zr]Zr-DFO-Her-TAT$_{(0\text{-}5)}$ internalised in SKBR3 cells after 48 h incubation, normalised for non-specific uptake derived from data presented in figure 5.*

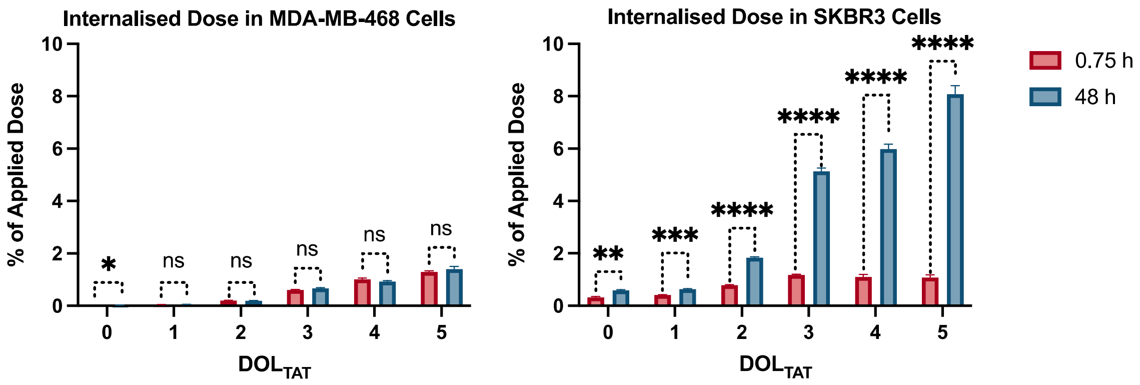

Figure S6: *Percentage of applied dose internalised in MDA-MB-468 (left) and SKBR3 (right) cells 0.75 (red) and 48 (blue) h after incubation of [^{89}Zr]Zr-DFO-Her-TAT$_{(0-5)}$ derived from data presented in figure 5.*

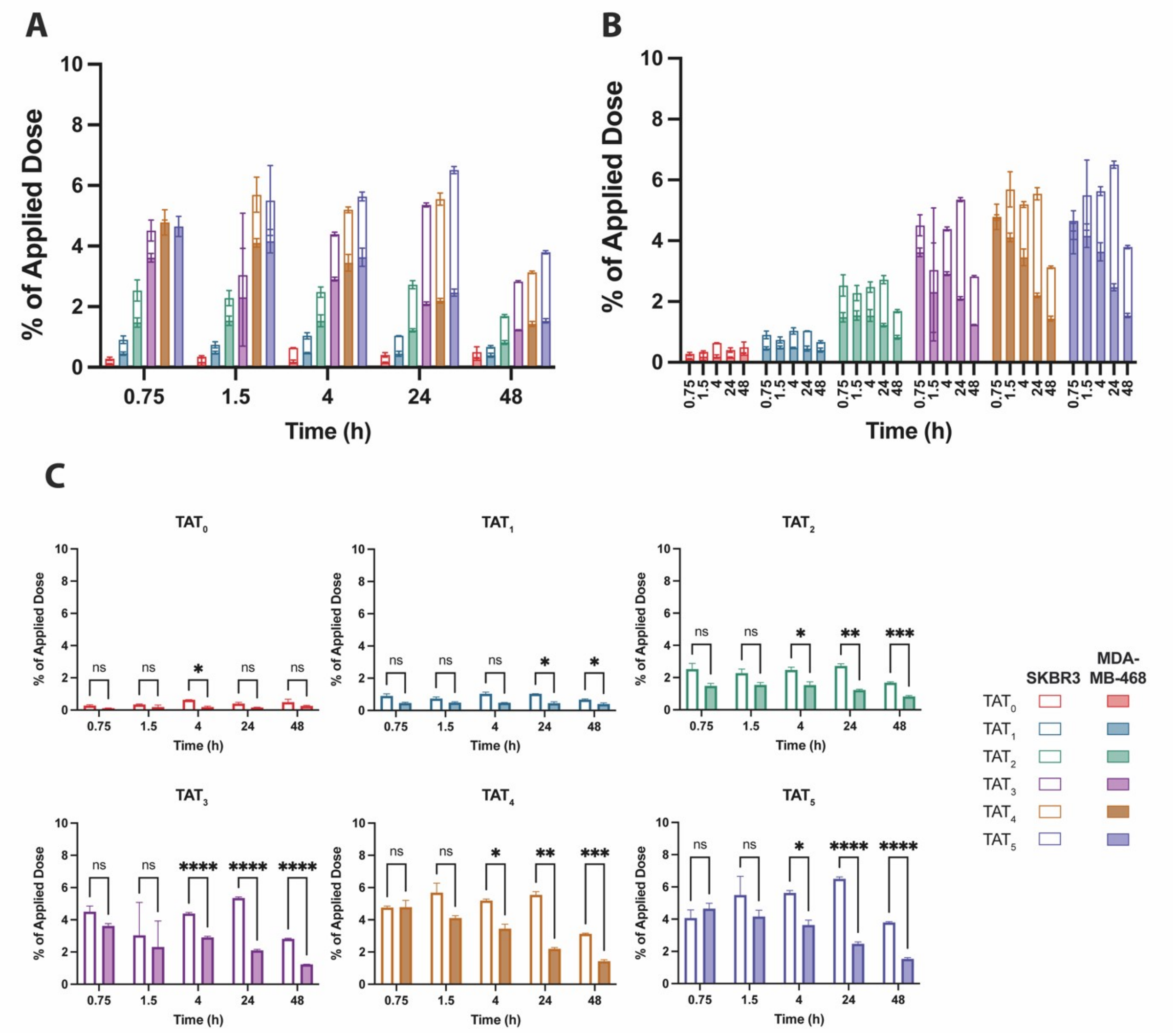

Figure S7: *Percentage of the applied dose that was membrane bound into SKBR3 (outlined bars) and MDA-MB-468 (filled bars) cells for each [^{89}Zr]Zr-DFO-Her-TAT$_{(0-5)}$ conjugate. (A) Overlaid membrane bound dose of each conjugate in SKBR3 and MDA-MB-468 cells at each time point grouped by timepoint. (B) Overlaid membrane bound dose of each conjugate in SKBR3 and MDA-MB-468 cells at each time point grouped by DOL$_{TAT}$. (C) Individual graphs for the membrane bound percentage of applied dose of each [^{89}Zr]Zr-DFO-Her-TAT$_{(0-5)}$ conjugate in SKBR3 and MDA-MB-468 cells.*

SUPPLEMENTARY TABLES

**Molar Excess of DBCO STP Ester**	**Average Yield ± SD (%)**	**Molar Excess of TAT-N_3**	**Average Yield ± SD (%)**
**1**	87.00 ± 8.04	10	80.15 ± 7.28
**2**	86.52 ± 2.34	10	82.44 ± 6.48
**3**	84.09 ± 1.66	10	79.31 ± 11.09
**4**	81.37 ± 4.73	10	83.39 ±13.76
**5**	75.27 ± 4.61	10	84.70 ± 5.44
**6**	68.07 ± 4.87	10	91.32 ± 4.25
**7**	63.78 ± 3.13	10	89.59 ± 7.21

Table S1: *Average yields (± standard deviation) for the synthesis of Her-DBCO and Her-TAT conjugates with DBCO degrees of labelling between 1 and 7.*

Her	Her-TAT$_{(1)}$		Her-TAT$_{(2)}$		Her-TAT$_{(3)}$		Her-TAT$_{(4)}$		Her-TAT$_{(5)}$	
[M+3]	[M+3]	Relative Intensity (a.u.)	[M+3]	Relative Intensity (a.u.)	[M+3]	Relative Intensity (a.u.)	[M+3]	Relative Intensity (a.u.)	[M+3]	Relative Intensity (a.u.)
49561.7	49604.7	92979	49619.1	69649	49659.0	42235	49727.7	14956	49753.2	5993
	50409.0	77469	50410.6	78653	50446.0	70158	50439.6	35327	50479.8	17188
	51151.7	28836	51166.3	48281	51244.1	67765	51244.1	44537	51315.5	24324
			52007.7		52017.5	43146	52033.8	41337	52072.3	23899
					52798.4	16700	52791.8	26270	52814.8	16998
							53548.6	12536	53678.1	8202

Table S2: *MALDI-TOF m/z [M+3] peaks and relative intensities for Her-TAT$_{(0-5)}$ conjugates.*

Time Point (h)	Her-TAT$_{(0)}$		Her-TAT$_{(1)}$		Her-TAT$_{(2)}$		Her-TAT$_{(3)}$		Her-TAT$_{(4)}$		Her-TAT$_{(5)}$	
	SKBR3	MDA-MB-468	SKBR3	MDA-MB-468	SKBR3	MDA-MB-468	SKBR3	MDA-MB-468	SKBR3	MDA-MB-468	SKBR3	MDA-MB-468
**0.75**	0.28	0.11	0.55	0.46	2.53	1.49	4.51	3.62	4.77	4.79	4.07	4.66
**1.5**	0.34	0.19	0.51	0.49	2.28	1.54	3.03	2.31	5.69	4.11	5.50	4.17
**4**	0.64	0.19	0.81	0.48	2.48	1.54	4.39	2.92	5.19	3.46	5.63	3.64
**24**	0.41	0.16	0.60	0.46	2.72	1.23	5.35	2.10	5.54	2.20	6.51	2.47
**48**	0.49	0.27	0.47	0.40	1.69	0.83	2.82	1.24	3.13	1.44	3.79	1.54

Table S3: *Average percentage of applied dose membrane bound of [^{89}Zr]Zr-DFO-Her-TAT$_{(0-5)}$ conjugates in SKBR3 and MDA-MB-468 cells.*

Time Point (h)	Her-TAT$_{(0)}$		Her-TAT$_{(1)}$		Her-TAT$_{(2)}$		Her-TAT$_{(3)}$		Her-TAT$_{(4)}$		Her-TAT$_{(5)}$	
	SKBR3	MDA-MB-468	SKBR3	MDA-MB-468	SKBR3	MDA-MB-468	SKBR3	MDA-MB-468	SKBR3	MDA-MB-468	SKBR3	MDA-MB-468
**0.75**	0.32	0.01	0.35	0.05	0.78	0.21	1.18	0.61	1.10	1.01	1.08	1.29
**1.5**	0.42	0.00	0.41	0.04	0.75	0.12	1.01	0.44	1.43	0.67	1.53	0.96
**4**	0.36	0.01	0.39	0.06	0.82	0.16	1.29	0.53	1.57	0.93	1.95	1.33
**24**	0.27	0.02	0.28	0.06	1.01	0.18	2.13	0.47	3.10	0.79	4.34	1.48
**48**	0.59	0.02	0.60	0.06	1.84	0.20	5.14	0.67	5.99	0.93	8.08	1.40

Table S4: *Average percentage of internalised applied dose of [^{89}Zr]Zr-DFO-Her-TAT$_{(0\text{-}5)}$ conjugates in SKBR3 and MDA-MB-468 cells.*

**Sample Name**	**DBCO Molar Excess**	**UV-Vis DOL_{TAT}**	**MALDI-TOF DOL_{TAT}**
**Her-$TAT_{(1)}$**	1	0.20	0.68
**Her-$TAT_{(2)}$**	2	1.37	0.89
**Her-$TAT_{(3)}$**	3	2.42	1.68
**Her-$TAT_{(4)}$**	4	3.90	2.38
**Her-$TAT_{(5)}$**	5	3.66	2.55

Table S5: *DOL_{TAT} of Her-$TAT_{(1-5)}$ conjugates as determined by UV-Vis and MALDI-TOF.*
